# Hydroxypropyl Cellulose as an Effective Binder for Low-Temperature Screen-Printed Porous Carbon Counter Electrodes for Indoor Dye-Sensitized Solar Cells

**DOI:** 10.3390/nano16161007

**Published:** 2026-08-17

**Authors:** Roberto Speranza, Elisa Morale, Filippo Sergiacomi, Angelica Bisceglie, Giorgio Mogli, Simone Martellone, Andrea Lamberti

**Affiliations:** 1 Department of Applied Science and Technologies, Politecnico di Torino, Corso Duca Degli Abruzzi 24, 10129 Torino, Italy; elisa.morale@studenti.polito.it (E.M.); filippo.sergiacomi@studenti.polito.it (F.S.); angelica.bisceglie@polito.it (A.B.); giorgio.mogli@polito.it (G.M.); simone.martellone@polito.it (S.M.); andrea.lamberti@polito.it (A.L.); 2 Center for Sustainable Future Technologies, Istituto Italiano di Tecnologia, 10129 Torino, Italy

**Keywords:** dye-sensitized solar cell, indoor photovoltaics, counter electrode, carbon composite, hydroxypropyl cellulose, low-temperature processing, screen printing

## Abstract

The development of indoor photovoltaic devices for powering Internet of Things (IoT) sensors requires low-cost and sustainable components, making dye-sensitized solar cells (DSSCs) an ideal candidate for artificial light harvesting. The counter electrode plays a critical role in transferring electrons and catalyzing the reduction in the redox electrolyte. However, the traditional use of expensive and scarce platinum (Pt) limits the cost-effective, large-scale commercialization of these devices. While carbon-based materials offer a highly porous, conductive, and abundant alternative, commercial carbon pastes frequently require energy-intensive high-temperature sintering. In this study, we propose a sustainable, low-temperature, and screen-printable carbon composite counter electrode (LoT-HPC) using bio-derived hydroxypropyl cellulose (HPC) as a highly effective binder. Rheological characterizations confirm that the formulated LoT-HPC ink possesses an ideal shear-thinning profile and rapid structural recovery, ensuring excellent printability and film homogeneity. By comparing the custom LoT-HPC composite against a commercial high-temperature screen-printed graphite paste (HT-Elco) and a standard sputtered Pt-FTO electrode, we demonstrate the structural and electrocatalytic advantages of this material. When integrated into full DSSC devices and evaluated under low indoor illumination (1000 lux), the LoT-HPC cell delivers a power conversion efficiency (PCE) of 14.8% and a high short-circuit current density of 103.9 µA cm^−2^. Furthermore, the custom device demonstrated exceptional operational stability, retaining 98.6% of its initial efficiency (from 14.8% to 14.6%) after 200 h of continuous light-soaking and J-V cycling under 1000 lux. Ultimately, the successful implementation of the HPC binder enables the low-temperature fabrication of sustainable carbon counter electrodes without the need for energy-intensive thermal treatments, presenting a highly scalable pathway for indoor DSSC manufacturing.

## 1. Introduction

Indoor photovoltaic cells (IPV) are bound to change the paradigm of how we power electronic devices, offering a sustainable pathway to strongly reduce or eliminate the reliance on disposable batteries. This technological shift is particularly relevant for the Internet of Things (IoT), an ecosystem of smart objects that continuously collect, process, and exchange information [1]. The exponential proliferation of these low-power electronic devices creates a massive and urgent demand for autonomous power sources. It is forecasted that the number of connected IoT devices will increase from 18 to 41 billion by 2035 [2]. While not all these devices can be realistically powered by indoor photovoltaics, such as power-demanding smart home appliances that remain wire-powered, a significant portion (approximately 25%, accounting for over 10 billion devices) use low or ultra-low power transmission protocols like Bluetooth, Zigbee, and LoRaWAN [2]. The energy demand of such devices is fully compatible with the current and future power outputs of indoor photovoltaic devices [3].

Indoor environments are intrinsically different from the standard outdoor conditions for which solar cells have been optimized for decades. The spectral composition of artificial illumination sources, such as fluorescent lamps or LEDs, lacks ultraviolet (UV) or infrared (IR) components, with photon emission concentrated almost entirely in the visible spectrum, between 320 nm and 780 nm [4]. For this reason, the optimal absorber material for indoor light harvesting should have an energy gap between 1.8 eV and 1.9 eV [5]. Therefore, third-generation photovoltaics, namely organic, perovskite and dye-sensitized solar cells (DSSCs), have emerged as the best candidates to penetrate the market of self-powered indoor devices. While each is characterized by its own peculiarities, these technologies share highly attractive features, including suitable energy gap, tuneable aesthetics and scalable fabrication at a low cost [6].

DSSCs are photo-electrochemical devices that mimic natural photosynthesis [7]. The standard architecture harvests light through the photo-induced oxidation of a dye (sensitizer) anchored to a mesoporous layer of TiO_2_ nanoparticles, which acts as the electron acceptor. Following dye excitation, the photogenerated electron is injected into the TiO_2_ matrix and diffuses toward the current collector, typically a transparent conductive oxide (TCO). The sensitized photoanode is in contact with an electrolyte containing a redox mediator. While the iodide/triiodide (I^−^/I_3_^−^) couple is the most traditional choice, novel mediators based on cobalt or copper coordination complexes have recently shown great promise in boosting device efficiency by significantly increasing the open-circuit voltage [8]. The redox mediator acts as an electron donor, regenerating the oxidized dye to its ground state. The device is completed by a counter electrode (CE), which includes a catalytic material necessary to facilitate the reduction of the oxidized redox mediator, therefore completing the electrical circuit and sustaining the photovoltaic process [9].

The counter electrode is a vital component, as its electrocatalytic activity directly determines the charge-transfer resistance at the electrode/electrolyte interface, impacting the overall efficiency of the cell. Conventionally, platinum (Pt) has been the preferred catalyst due to its exceptional electrocatalytic activity and superior electrical conductivity [10]. However, the high cost, rarity, and susceptibility to corrosion and degradation in iodine-based electrolytes severely restrict its practical viability for the large-scale commercialization of DSSCs [11]. In this framework, carbonaceous materials (e.g., carbon black, activated carbon, graphite, and carbon nanotubes) have gained immense interest as noble-metal-free alternatives. These carbon-based materials offer low cost, excellent chemical stability, high surface area, and good electrocatalytic activity toward redox couple reduction [12,13,14].

For the commercial mass production of DSSCs, screen printing is considered one of the most viable and scalable deposition techniques [15]. Formulating screen-printable carbon inks typically requires the incorporation of polymeric binders to achieve proper rheology and ensure adequate mechanical adhesion to the conductive substrate [16]. However, conventional binders, such as ethyl cellulose, polyvinylidene fluoride (PVDF), or polytetrafluoroethylene (PTFE), are electrically insulating. If left intact, these polymers form a barrier to electron transport and mask the catalytic carbon sites [17]. To prevent this, printed carbon layers generally need a high-temperature firing step, often between 400 °C and 500 °C, to thermally decompose the binder and establish a highly conductive, interconnected carbon network. For instance, highly conductive commercial screen-printable carbon pastes typically require firing at 400 °C for approximately 40 min to yield an active and conductive layer [18].

The necessity of high-temperature thermal treatments increases energy consumption during manufacturing and strictly precludes the use of lightweight, flexible polymeric substrates, which degrade rapidly at such elevated temperatures. To bypass these limitations and advance the sustainability of IPVs, the development of low-temperature-processed carbon CEs is highly desirable. In this context, hydroxypropyl cellulose (HPC) presents a compelling alternative as a sustainable, fluorine-free, and bio-derived binder [17]. While HPC has recently been demonstrated as a highly effective and stable aqueous binder for supercapacitor electrodes, its integration into screen-printable inks for DSSC counter electrodes remains completely unexplored. Transitioning from aqueous supercapacitor slurries to screen-printed photovoltaic catalytic layers introduces fundamental differences in both processing and operating electrochemistry. First, while supercapacitor electrodes are processed in water-based slurries, formulating homogeneous and screen-printable carbon inks for DSSCs requires a specialized organic solvent system, achieved here for the first time using a mixture of ethanol and terpineol. Second, and more crucially, supercapacitors operate under non-faradaic double-layer charge storage in supporting electrolytes. In contrast, a DSSC counter electrode operates under drastically different and chemically aggressive conditions, as it must continuously catalyze the faradaic reduction in the highly corrosive iodide/triiodide redox couple. Consequently, this study represents the first trial of HPC as a sustainable, fluorine-free binder in DSSC counter electrodes, decoupling the impact of the intact polymer network on charge percolation and catalytic performance.

Bypassing the high-temperature firing step inevitably leaves the insulating HPC binder intact within the carbon matrix, which typically increases the charge-transfer resistance and may reduce the absolute electrocatalytic performance under standard 1 Sun illumination, where very high values of current densities are photogenerated [13]. However, this limitation is intrinsically mitigated under indoor lighting conditions. In indoor environments (e.g., 100–1000 lux), the irradiance is orders of magnitude lower than standard sunlight, resulting in photogenerated currents in the range of microamperes rather than milliamperes [19]. At such extremely low current densities, the counter electrode operates at very low overpotentials. Consequently, the detrimental impact of a higher charge-transfer resistance on the fill factor and overall photovoltaic efficiency is drastically reduced. Therefore, while indoor counter electrodes must still exhibit sufficient catalytic activity to sustain these photogenerated currents, the relaxation of these kinetic requirements significantly elevates the importance of material sustainability, low-temperature processability, stability, and cost-effectiveness. This shift in design priorities is crucial for the targeted integration of indoor DSSCs into consumer electronics and Internet of Things (IoT) networks, where high manufacturing costs or excessive processing complexity would otherwise render such large-scale commercial application economically unviable.

In this work, we report the first demonstration of HPC as a sustainable binder in DSSCs. We developed a low-cost, screen-printable carbon ink composed of activated carbon (AC), carbon black (CB), HPC and terpineol as the solvent. Unlike conventional carbon pastes that necessitate energy-intensive, high-temperature firing, our formulated ink requires only a low-temperature drying process at 100 °C for 15 min. We demonstrate that this low-temperature HPC-based carbon counter electrode, while exhibiting lower absolute performance than the commercial reference paste, delivers highly comparable performance under the low illuminance levels typical of indoor environments. This approach offers a highly sustainable, energy-efficient, and potentially flexible pathway for the fabrication of DSSC counter electrodes tailored specifically for indoor applications.

## 2. Materials and Methods

### 2.1. Materials

Unless otherwise specified, all the materials were used as received without further purification or additional treatments. Fluorine-doped tin oxide (FTO)-coated glass (7 Ω/sq), thermoplastic sealing film (Meltonix, 60 µm), Ruthenizer 535-bisTBA (N719 dye), TiO_2_ blocking layer paste (Ti-Nanoxide BL/SP), transparent TiO_2_ nanoparticle paste (Ti-Nanoxide T/SP) and graphite/carbon black paste (Elcocarb B/SP) were acquired from Solaronix (Aubonne, Switzerland). Activated carbon (YP-50 F) was received from Kuraray (Tokyo, Japan) and carbon black (C65) was acquired from Imerys (Paris, France). Hydroxypropyl cellulose (HPC, Mw 370,000), α-terpineol, sodium iodide (NaI), iodine (I_2_), 4-tert-butylpyridine (TBP) and γ-valerolactone were purchased from Sigma-Aldrich and Merck (Darmstadt, Germany).

### 2.2. Preparation of the Screen-Printable Ink and Counter Electrodes

The formulation of the screen-printable carbon ink was adapted from our previous work, using a solid-state mass composition of 85 wt% AC, 10 wt% CB, and 5 wt% HPC. To prepare the ink, the binder was initially dissolved in ethanol under continuous mechanical agitation to ensure complete polymer solvation. Subsequently, the highly conductive CB nanoparticles and the high-surface-area AC were sequentially dispersed into the mixture, with vigorous agitation maintained until a homogeneous suspension was achieved. Following this, α-terpineol was introduced into the mixture to serve as the primary viscous vehicle for the screen-printing process. The dispersion was then left under continuous mechanical stirring overnight in an open vessel to facilitate the complete evaporation of the highly volatile ethanol. The optimized formulation contained a final α-terpineol content of 71 wt%.

The custom ink was compared with the Elcocarb B/SP commercial paste acquired from Solaronix. Both inks were screen-printed with a 43–80 mesh on top of FTO-glass. The FTO-coated glasses were first cleaned in an ultrasonic bath using a sequential sequence of deionized water with detergent, acetone, and ethanol for 15 to 30 min, followed by drying on a hot plate at 100 °C. After each print, the low-temperature, HPC-based counter electrodes (LoT-HPC) were dried at 100 °C for 15 min on a hot plate. Also, the high-temperature, Elcocarb counter electrodes (HT-Elco) were dried at 100 °C for 15 min after each printed layer and then fired at 400 °C for 40 min with a thermal ramp of 120 °C/h. The screen-printed counter electrodes were compared with a reference Pt electrode (Pt-FTO) prepared by depositing a thin layer of Pt onto the FTO glasses via sputtering (using a Q150T ES sample preparation system, Quorum Technologies, Laughton, UK, deposition current of 30 mA, deposition time of 30 s). The active area of the counter electrodes was a square of 12 × 12 cm (1.44 cm^2^).

### 2.3. Photoanode Preparation and DSSC Assembly

The FTO-coated glasses were first cleaned in an ultrasonic bath using a sequential sequence of deionized water with detergent, acetone, and ethanol for 15 to 30 min, followed by drying on a hot plate at 100 °C. A compact TiO_2_ blocking layer was deposited on the FTO substrates by screen printing a layer of Ti-Nanoxide BL/SP paste, which was dried at 100 °C for 10 min and then fired at 500 °C for 40 min. Subsequently, the mesoporous photoanodes were fabricated by screen-printing three layers of transparent TiO_2_ nanoparticle paste. The printed photoanodes were gradually heated to 475 °C and sintered for 30 min to interconnect the TiO_2_ particles. Once cooled to approximately 70–80 °C, the photoanodes were immersed in a 0.3 mM solution of N719 dye in ethanol for 16 to 18 h to ensure complete sensitization. The active area of the deposited photoanodes was 1.44 cm^2^, while their thickness was measured to be between 8 μm.

The sensitized photoanodes and the respective counter electrodes were assembled in a sandwich configuration and sealed using two thermoplastic spacers (Meltonix) via a hot press. An iodine-based liquid electrolyte (0.45 M NaI, 0.056 M I_2_, and 0.55 M TBP) was injected into the interelectrode space through pre-drilled holes, which were subsequently sealed to prevent leakage. The electrolyte composition was replicated from our previous work, using γ-valerolactone as solvent, which proved to be an excellent sustainable alternative for indoor DSSCs [20].

### 2.4. Material and Electrode Characterization

To evaluate the relationship between the number of printed layers, thickness, and sheet resistance, multiple carbon layers were printed onto non-conductive microscope glass slides. Sheet resistance measurements were conducted via a four-point probe method using a Jandel HM21 test unit (Jandel Engineering Limited, Leighton Buzzard, UK). The surface morphology of the samples was examined using a SUPRA 40 (Zeiss, Oberkochen, Germany) field-emission scanning electron microscope (FESEM) operating at 5 kV accelerating voltage and 20 μm aperture.

Rheological measurements were performed using an Anton Paar Physica MCR 302 rheometer (Anton Paar, Graz, Austria) equipped with a parallel plate geometry (25 mm diameter). All measurements were conducted at 25 °C with a gap of 0.2 mm. The flow behaviour of the samples was investigated by measuring viscosity as a function of shear rate over the range 0.01–10^4^ s^−1^. This test was used to evaluate shear-dependent viscosity and to assess the presence of shear-thinning behaviour. Amplitude sweep tests were carried out at a constant frequency of 1 Hz, varying the strain amplitude from 0.1% to 1000%. The storage modulus (G′) and loss modulus (G″) were monitored to determine the linear viscoelastic region (LVR) and to evaluate the structural stability of the samples under increasing deformation.

Thixotropic behaviour was then assessed using a three-interval thixotropy test (3ITT) at 1 Hz. The test consisted of three consecutive steps:

- First interval (rest condition): low strain (0.5%, within LVR) to measure the undisturbed structure of the ink;
- Second interval (high deformation): high strain (100%, outside LVR) to simulate processing conditions and induce ink structural breakdown;
- Third interval (recovery): low strain (0.5%, within LVR) to monitor structural rebuilding over time.

Both viscoelastic (G′) and viscosity recovery were recorded during the test.

Thermogravimetric analyses (TGA) were carried out through a thermogravimetric analyzer (Mettler Toledo, Greifensee, Switzerland). Sample weight changes were monitored in the 25–800 °C interval, using a heating rate of 10 °C min^−1^ under air atmosphere (50 mL min^−1^) and nitrogen as protective gas (20 mL min^−1^).

To simulate the drying process of the inks, samples with a nominal mass of 19 mg were heated from 25 °C to 100 °C at a heating rate of 10 °C min^−1^, followed by an isothermal step at 100 °C for 100 min. This protocol was employed to evaluate the rate of solvent evaporation.

Specific surface area was measured at 77 K by N_2_ sorption porosimetry using an ASAP2020 Plus (Micromeritics, Norcross, GA, USA). The thermally treated sample was heated to 250 °C under vacuum for 180 min, with a ramp rate of 10 °C min^−1^ to remove the residual solvent.

### 2.5. Counter Electrodes and DSSCs Characterization

The electrochemical properties of the counter electrodes were investigated in a three-electrode cell, including the counter electrode as a working electrode, a Pt spring as a counter electrode, and a Pt wire as a pseudo-reference electrode. The electrolyte composition for the 3-electrode measurements was adapted from the literature, and it was 10 mM NaI, 1 mM I_2_ and 0.1 M NaClO_3_ in acetonitrile [21,22]. Cyclic voltammetry was performed at a scan rate of 50 mV s^−1,^ while Electrochemical Impedance Spectroscopy (EIS) spectra were recorded in the frequency range from 100 kHz down to 100 mHz, with a small signal amplitude of 10 mV. Full DSSCs were investigated using EIS and linear polarization measurements. EIS spectra were recorded in the dark, applying a constant-voltage bias of 0.6 V and scanning a frequency range from 100 kHz down to 100 mHz, with a small signal amplitude of 10 mV. The scan speed for the current density–voltage (J–V) characteristics of the assembled DSSCs was 20 mV s^−1^. A Versatile Multichannel Potentiostat, VMP 3, provided by BioLogic (Seyssinet-Pariset, France) and an Autolab PGSTAT128 potentiostat equipped with an FRA32M module were used to carry out electrochemical characterizations (Metrohm, Herisau, Switzerland). To evaluate the photovoltaic performance under different lighting conditions, the devices were characterized using a calibrated LightBox XL indoor light simulator (Lightricity Ltd., Oxford, UK). The LightBox XL is equipped with an 11 × 11 array of white LEDs capable of providing highly uniform illumination with a correlated colour temperature (CCT) of 3000 K, a colour rendering index of 70, and a uniformity of 95%. The devices were tested at illuminance levels of 100, 500 and 1000 lux according to the guidelines recently released in the IEC 62607-7-2 technical specification [23]. The corresponding irradiance values were 0.03, 0.15 and 0.3 mW cm^−2^, respectively.

## 3. Results

### 3.1. Rheology, Printability, Thermal and Morphological Characterization of the Ink

To ensure a successful deposition of the carbon catalyst via screen printing, the formulated ink must behave as a two-phase system exhibiting non-Newtonian, shear-thinning and thixotropic behaviour [24]. This rheological profile is crucial for ensuring that the ink’s viscosity rapidly decreases under the high shear stress applied by the squeegee, allowing the carbon suspension to smoothly flow through the patterned mesh. Upon removal of the shear stress, the viscosity must recover quickly enough during the levelling phase to maintain a high resolution of the printed pattern and prevent uncontrolled spreading over the substrate.

**Figure 1:**
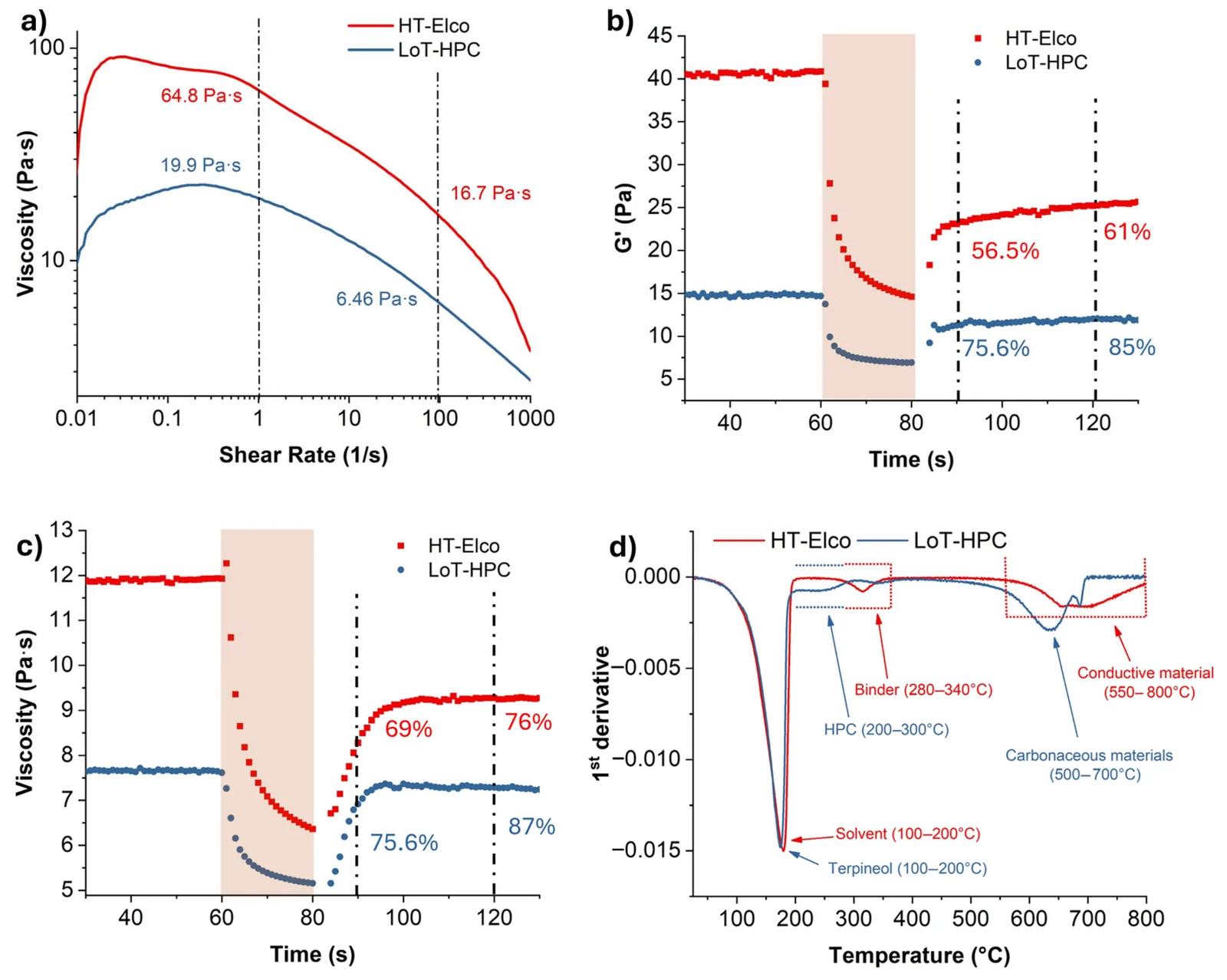
Rheological properties of the custom low-temperature carbon ink (LoT-HPC) and the commercial high-temperature carbon paste (HT-Elco): (**a**) Viscosity as a function of shear rate. Viscosity values of the inks at 1 s^−1^ and 100 s^−1^ are reported. (**b**,**c**) Three-interval thixotropy test (3iTT) displaying the time-dependent recovery profiles of (**c**) the storage modulus (G′) and (**d**) the dynamic viscosity. The light orange rectangle highlights the interval where a 100% strain was applied to simulate the screen-printing process. Percentage recovery values for G′ and viscosity at 10 s and 40 s (plateau) are labelled close to the curves to highlight the structural rebuilding kinetics of each ink. Thermal-gravimetric analysis of the inks: (**c**) First derivative of the weight-loss curves of the inks over the temperature range 25–800 °C. The labels indicate the components of the inks and their degradation temperatures corresponding to the curve peaks.

Rheological characterizations confirmed that both the custom LoT-HPC ink and the commercial HT-Elco paste possess this ideal shear-thinning profile (Figure 1a). At rest (1 s^−1^), the commercial HT-Elco paste exhibits a viscosity of approximately 64.8 Pa·s, while the LoT-HPC ink shows a lower rest viscosity of 19.9 Pa·s. At a shear rate of 100 s^−1^, which simulates the shear forces during the screen-printing deposition phase, the viscosities drop to 16.7 Pa·s and 6.46 Pa·s, respectively, facilitating optimal flow through the mesh. The lower viscosity of LoT-HPC ink perfectly lies in the 1–10 Pa·s viscosity range typically indicated in the literature for screen printing, suggesting superior penetration through the finer meshes compared to the commercial counterpart [25]. Amplitude sweep tests, conducted to investigate the viscoelastic properties and the structural changes in the inks under deformation, revealed that the HT-Elco paste maintains a balanced viscoelastic behaviour with similar G′ and G″ moduli over the linear viscoelastic range (LVR), comprised between 0 and 10% strain (Figure S1a). In contrast, the LoT-HPC ink displays a more liquid-like behaviour across the tested strain range (G″ > G′), with a similar LVR. This behaviour enhances LoT-HPC ink ductility, effectively reducing the required squeegee pressure and facilitating higher deposition speeds. However, this increased fluidity may lead to sagging and bleeding, potentially compromising edge resolution compared to the more balanced viscoelastic profile of the commercial HT-Elco paste [25].

Importantly, this drawback is mitigated by the LoT-HPC ink’s superior structural recovery kinetics, which ensures rapid stabilization after printing. A three-interval thixotropy test (3iTT) was performed to simulate the real application of the ink, evaluating the recovery phase of the inks (Figure 1b,c). The LoT-HPC ink demonstrated superior and rapid structural rebuilding, recovering 88.8% of its viscosity and 75.6% of its storage modulus (G′) within just 10 s of the stress removal. The HT-Elco paste exhibited slower recovery rates of 69.0% and 56.5%, respectively, in the same timeframe. The thixotropic advantage of the LoT-HPC ink is further evidenced at the plateau region, after approximately 20 s. With a recovery of 87% for G’ and 95% for viscosity, compared to 61% and 76% for HT-Elco, the custom ink ensures a more complete structural stabilization. This characteristic is vital for maintaining pattern resolution while allowing for the effective levelling of the ‘mesh signs’ left during printing, creating a continuous and homogeneous film.

The thermal properties and precise composition of the formulated inks were verified via thermogravimetric analysis (TGA) under an air atmosphere (Figure S1b). The first derivative of TGA profiles (Figure 1d) revealed three distinct weight-loss stages for both inks: solvent volatilization between 100 °C and 200 °C, binder decomposition between 200 °C and 400 °C, and carbon oxidation between 500 °C and 800 °C.

During the initial step, the LoT-HPC ink exhibited a sharp DTG peak centred around 175 °C, corresponding to a weight loss of approximately 60% due to terpineol evaporation. A subsequent shoulder is evident between 200 °C and 350 °C, likely representing the combined degradation of the HPC binder (which constitutes 1.45 wt% of the total ink) and the release of residual bound terpineol trapped within the carbon/polymer matrix. This process leaves approximately 28% of carbonaceous material residue, degraded at higher temperatures.

In contrast, the HT-Elco paste showed a lower solvent weight loss of 30% and a distinct binder degradation peak that persists up to approximately 400 °C. This reflects a significantly higher binder presence (approximately 5 wt% of the total weight) compared to the custom ink. These findings justify the requirement for a higher firing temperature (400 °C) for the commercial paste to ensure complete binder removal and establish optimal electrical conductivity, whereas the LoT-HPC ink is compatible with low-temperature drying at 100 °C due to its lower binder content and rapid solvent release.

**Figure 2:**
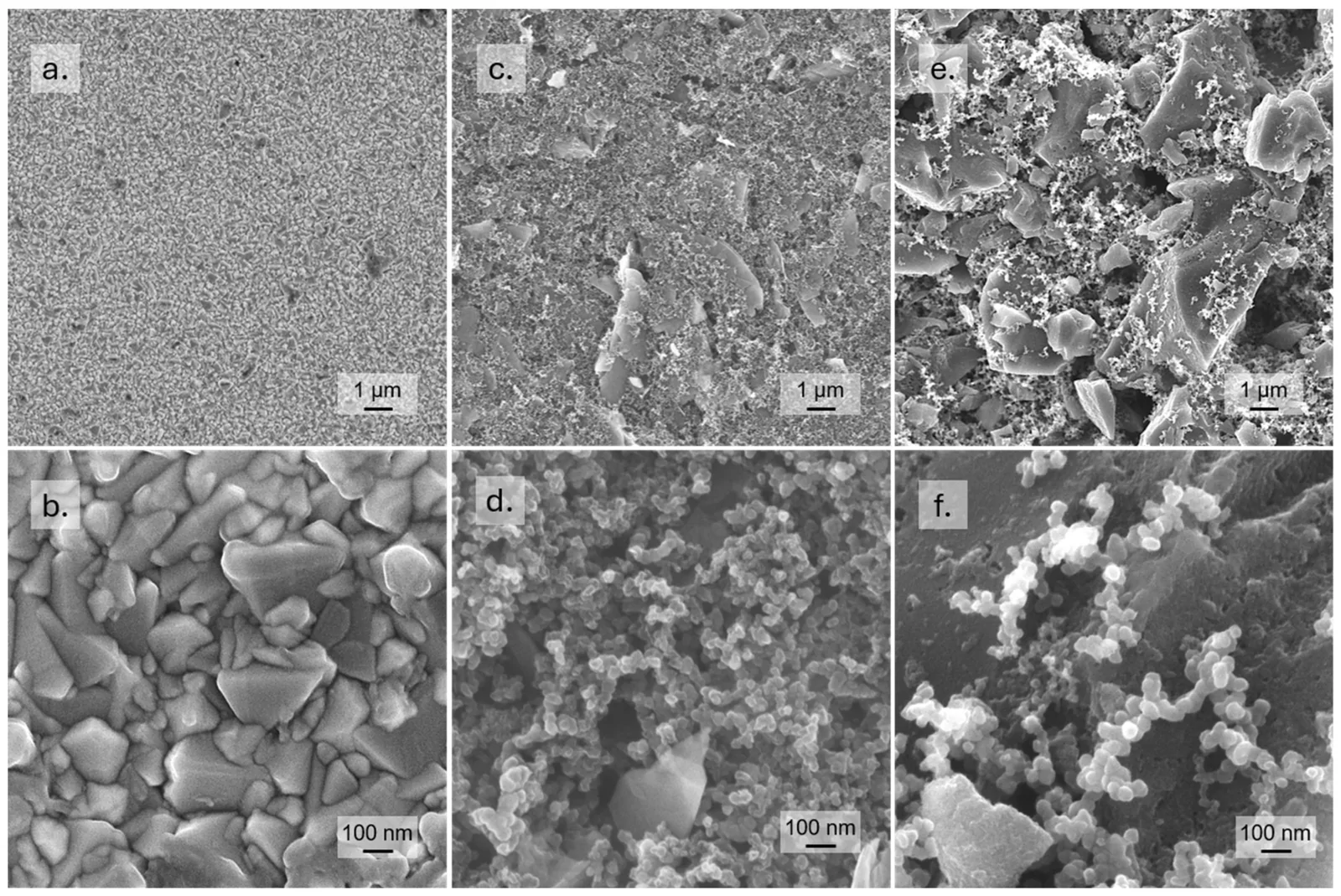
FESEM micrographs at different magnifications of (**a**,**b**) Pt-covered ITO; (**c**,**d**) commercial HT-Elco graphite-based ink; (**e**,**f**) LoT-HPC carbon ink.

The FESEM analysis highlighted clear differences in surface morphology among the investigated counter electrodes. For the sputtered Pt-FTO reference electrode, the low-magnification image shows the typical rough surface of bare FTO, which consists of pyramid-like crystals. The corresponding high-magnification view reveals a uniform layer of sputtered Pt nanoparticles distributed over the larger FTO crystals, with no visible clusters or aggregates (Figure 2a,b).

The HT-Elco fired at 400 °C presents, at low magnification, a highly heterogeneous and compact layer composed of larger graphite flakes mixed with smaller carbon black particles. At high magnification, no binder residue is evident, as expected after the thermal decomposition of organic binders during the high-temperature firing step (Figure 2c,d). This electrode exhibits a relatively flat and dense topography. The highly interconnected network of randomly distributed carbon black nanoparticles over the graphite flakes appears to be the main source of porosity, providing a reasonably high electrocatalytic surface area.

In contrast, the custom LoT-HPC carbon ink dried at 100 °C exhibits a rougher composite structure. The low-magnification image highlights a surface with evident macropores, likely due to the relatively small ratio of carbon black to activated carbon. Although FESEM does not allow a direct assessment of meso- or microporosity, the high-magnification image reveals a more complex porous texture of the high-surface-area activated carbon particles. This hierarchical pore structure is likely to provide a significantly higher electrocatalytic surface area compared to the non-porous graphite flakes in the HT-Elco paste, though this improvement comes at the cost of reduced electrical conductivity, as disordered activated carbon is less conductive than highly oriented graphite. Although the 100 °C drying step falls well below the 200–300 °C thermal degradation range of HPC, ensuring the binder remains fully preserved, no visible polymeric aggregates are observed in the high-magnification image. The absence of visible HPC traces or macroscopic polymer agglomerates, with activated carbon and carbon black particles clearly visible, demonstrates excellent dispersion and homogeneity of the formulated ink. Despite not being clearly identifiable under FESEM imaging, the intact polymer network ensures mechanical cohesion of the catalytic layer and stable adhesion to the FTO substrate, eliminating the need for an energy-intensive calcination step.

The structural integrity provided by the respective binders allows for precise control of the final electrode thickness by varying the number of sequentially printed layers. As the number of layers increases, the overall dry film thickness increases, establishing a carbon network that effectively reduces the sheet resistance of the electrodes. The relationship between the number of printed layers, the corresponding dry thickness, and the resulting sheet resistance for both the custom LoT-HPC ink and the commercial HT-Elco paste is summarized in Table 1. A single printed layer of the LoT-HPC ink yields a substantially thicker film (28.7 µm) compared to the HT-Elco paste (15.5 µm), reflecting the different solid contents and rheological levelling behaviour of the two formulations. Furthermore, the sheet resistance of the custom LoT-HPC electrodes remains much higher than that of the commercial HT-Elco electrodes across all printed layers, with the lowest values obtained for three printed layers, equal to 206 Ω/sq versus 7.6 Ω/sq for the LoT-HPC and HT-Elco, respectively. This expected difference is a direct consequence of the presence of the low-conductive AC particles instead of the graphite flakes included in the HT-Elco paste.

The specific surface area (SSA) of the custom ink used to obtain the counter electrode was studied by nitrogen sorption porosimetry at 77 K. It is interesting to note that the effect of thermal treatment was also assessed. The untreated electrode material exhibits a type II isotherm according to IUPAC classification (Figure S6) [26].

The very low nitrogen adsorption across the entire pressure range indicates that the accessible microporosity is negligible and that most of the internal surface area is inaccessible to nitrogen prior to heat treatment.

The thermal treatment at 250 °C for 3 h completely changes the picture. Indeed, the isotherm changes from type II to type I. The steep increase in adsorption at very low *p*/*p*° values is due to greater adsorbent–adsorbate interactions within the narrow micropores, which cause the micropores to fill at very low *p*/*p*° values. This interpretation is confirmed by the Non-Local Density Functional Theory (NLDFT) analysis (inset in Figure S6), where the pore distribution of this type of material can be observed. A slight increase in the amount of adsorbed gas is visible even at high relative pressures, attributable to interparticle voids [27].

The increase in SSA due to microporosity is huge; indeed, the BET approach (applied according to Roquerol’s criteria for microporous materials) reveals that the value rises from 22 m^2^g^−1^ to 1123 m^2^g^−1^. Furthermore, analysis of the t-plot indicates that approximately 75% of the total BET surface area corresponds to micropores, while the percentage of micropores in the untreated sample is almost null. The possible explanation for this evident SSA increase is primarily the evaporation of residual terpinol, which occludes the micropores. This is also confirmed by the TGA results, which show that the solvent evaporates mostly in the 100–200 °C range; the TGA indicates that a minor contribution arising from the partial decomposition of the binder is possible.

### 3.2. Counter Electrodes Electrochemical Characterization

**Figure 3:**
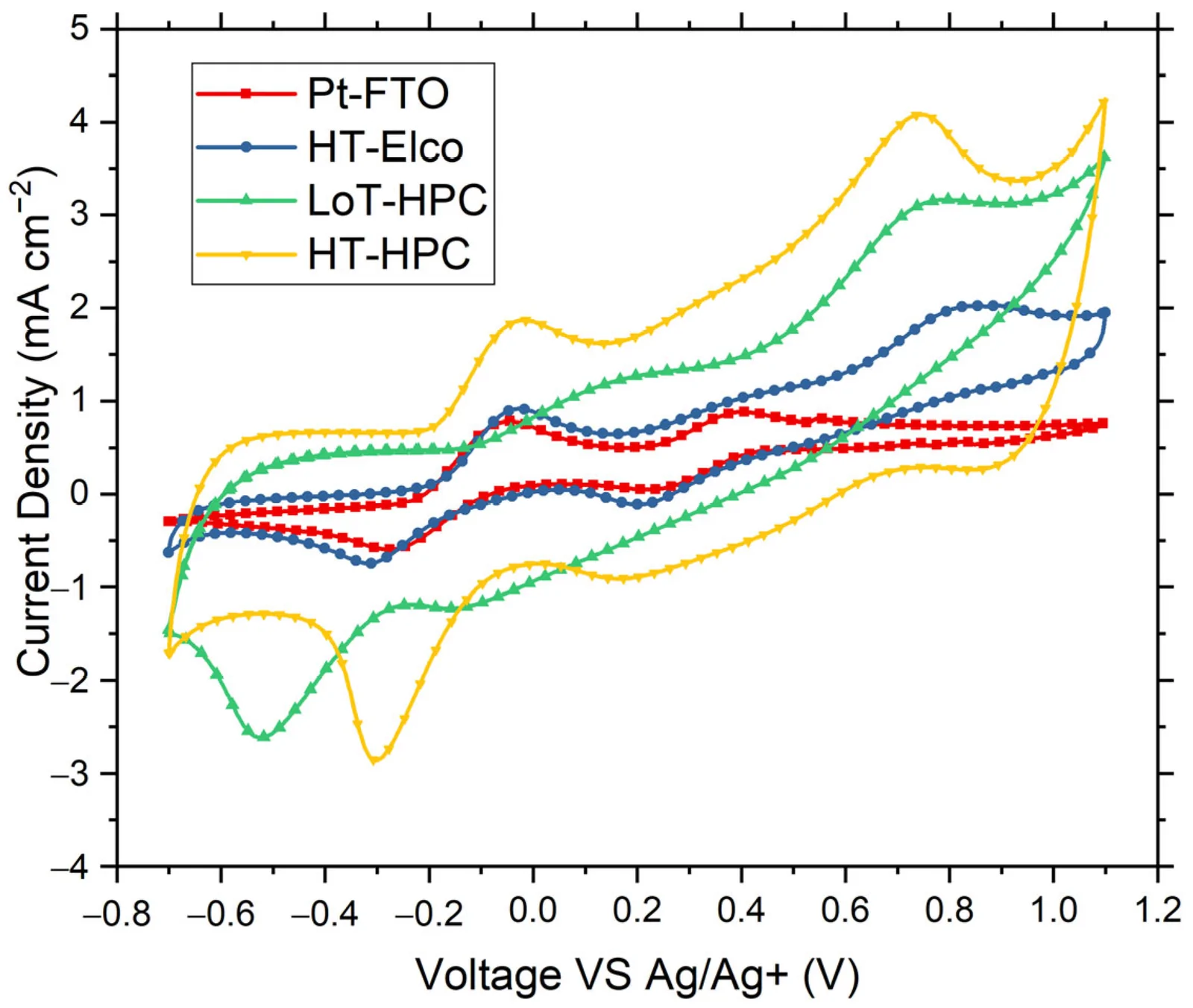
Cyclic voltammograms (CV) in half-cell configuration recorded at a scan rate of 10 mV s^−1^ of the reference Pt-FTO, the commercial HT-Elco, the custom LoT-HPC electrode and the custom electrode treated for 2 h at 250 °C in vacuum (HT-HPC), evaluated in a three-electrode configuration. All measurements were conducted in a dilute iodide/triiodide electrolyte (10 mM NaI, 1 mM I_2_, 0.1 M NaClO_4_ in acetonitrile) employing a Ag/Ag+ reference electrode and a Pt spring counter electrode.

To evaluate the intrinsic electrocatalytic activity of the prepared materials toward the iodide/triiodide (I^−^/I_3_^−^) redox system, cyclic voltammetry (CV) was conducted in a three-electrode configuration (Figure 3). Qualitative analysis of the CV profiles reveals distinct interfacial and charge-transport behaviours for the investigated counter electrode materials. The reference Pt-FTO electrode exhibits the classical, highly reversible voltammetric signature of the iodide/triiodide redox couple in acetonitrile, characterized by two well-defined pairs of anodic and cathodic peaks [28,29]. The first pair corresponds to the oxidation/reduction in the I^−^/I_3_^−^ couple, while the second, at more positive potentials, is associated with the I_3_^−^/I_2_ process. Additionally, Pt-FTO displays a negligible double-layer charging capacitive current, as expected for a flat, non-porous surface [30].

For the HT-Elco electrode, the characteristic redox peaks remain clearly visible, although with a moderately broader peak-to-peak separation (ΔE_p_) indicating slightly slower intrinsic electron transfer kinetics compared to Pt. Nevertheless, the mesoporous nature of this carbon network, which enhances the electroactive surface area, allows the HT-Elco electrode to provide comparable peak current density values, confirming that, at a matching geometric area, the HT-Elco layer (provided the complete thermal decomposition of organic binders and terpineol solvent at 400 °C) delivers a competitive electrocatalytic activity.

On the other hand, the low-temperature composite electrode (LoT-HPC, dried at 100 °C for 15 min) shows a much larger capacitive background, which is the typical fingerprint of double-layer charging (EDLC) within the massive microporous network of the activated carbon. However, the faradaic peaks for the LoT-HPC electrode appear depressed and distorted, showing a pronounced slope that is characteristic of high ohmic drop and charge-transport limitations. This resistive distortion is particularly clear at high anodic potentials, generating a strong asymmetry between the forward and reverse sweeps. This behaviour is most likely linked to the low-temperature drying process, which is insufficient to evaporate the high-boiling-point terpineol solvent, as observed from the previously reported TGA measurement, leaving a combination of trapped solvent molecules to clog the micropores and passivate the active catalytic sites.

Because the primary function of a counter electrode in a dye-sensitized solar cell is to catalyze the reduction of triiodide to iodide, the cathodic peak located in the lower potential region (corresponding to the reaction I_3_^−^ + 2e^−^ → 3I^−^) is the principal indicator of device-relevant electrocatalytic performance. The true faradaic peak currents should be extracted via baseline extrapolation to eliminate the EDLC charging background inherent to highly porous carbonaceous networks [31]. Unfortunately, especially for the LoT-HPC electrode, a clear identification of the capacitive baseline is not possible; therefore, the catalytic activity was evaluated by examining the peak-to-peak voltage separation mentioned before. The Pt-FTO electrode exhibits a ΔE_p_ of 0.241 V while the commercial HT-Elco paste displays a higher ΔE_p_ of 0.287 V. Finally, the custom LoT-HPC composite shows a significantly higher ΔE_p_ of 0.759 V, which further suggests strong ohmic losses limiting the reversibility of the reaction. Furthermore, as previously indicated by the BET measurements, the low-temperature treatment is insufficient to completely evaporate the terpineol solvent, which severely diminishes the specific surface area (SSA) by clogging the porous network. Nevertheless, it is evident that at a matching geometric area, the LT-HPC electrode still provides a much larger electroactive surface area compared to the other two materials, yielding a substantially higher cathodic peak current density associated with the reduction of triiodide. To decouple and quantify the detrimental effect of the residual terpineol, an identical composite electrode (denoted as HT-HPC) was subjected to a thermal treatment at 250 °C for 2 h in a vacuum oven, aiming at the complete removal of the trapped solvent. The comparison between the CV of the LT-HPC and HT-HPC electrodes (Figure 3) demonstrates the expected outcomes upon terpineol removal: an overall enhancement of the current response and, most importantly, a drastic suppression of the resistive distortions. Specifically, the ΔE_p_ drops significantly from 0.759 V to 0.283 V, reaching a value comparable to that of the HT-Elco electrode. This result definitively confirms that the incomplete solvent evaporation in the LT-HPC sample limits the electrode’s performance, affecting both the electronic transport pathways and the accessibility of the catalytic sites. However, it must be noted that the massive increase in SSA observed via nitrogen sorption, following the complete terpineol removal, does not translate into an electrochemical enhancement of the exact same magnitude. This discrepancy arises because nitrogen molecules can probe ultra-fine micropores that may not be easily accessible to the much bulkier solvated electrolyte ions due to steric hindrance [32]. Indeed, considering that the bare triiodide ion has an approximate dimension of 3 Å in diameter and 6 Å in length, considering also the solvation sphere, pores below the low-mesoporous range may act as a physical barrier [12]. Consequently, not the entire SSA detected by BET gas sorption actively contributes to the formation of the electrochemical double layer or to the electrocatalytic reduction of the redox mediator.

**Figure 4:**
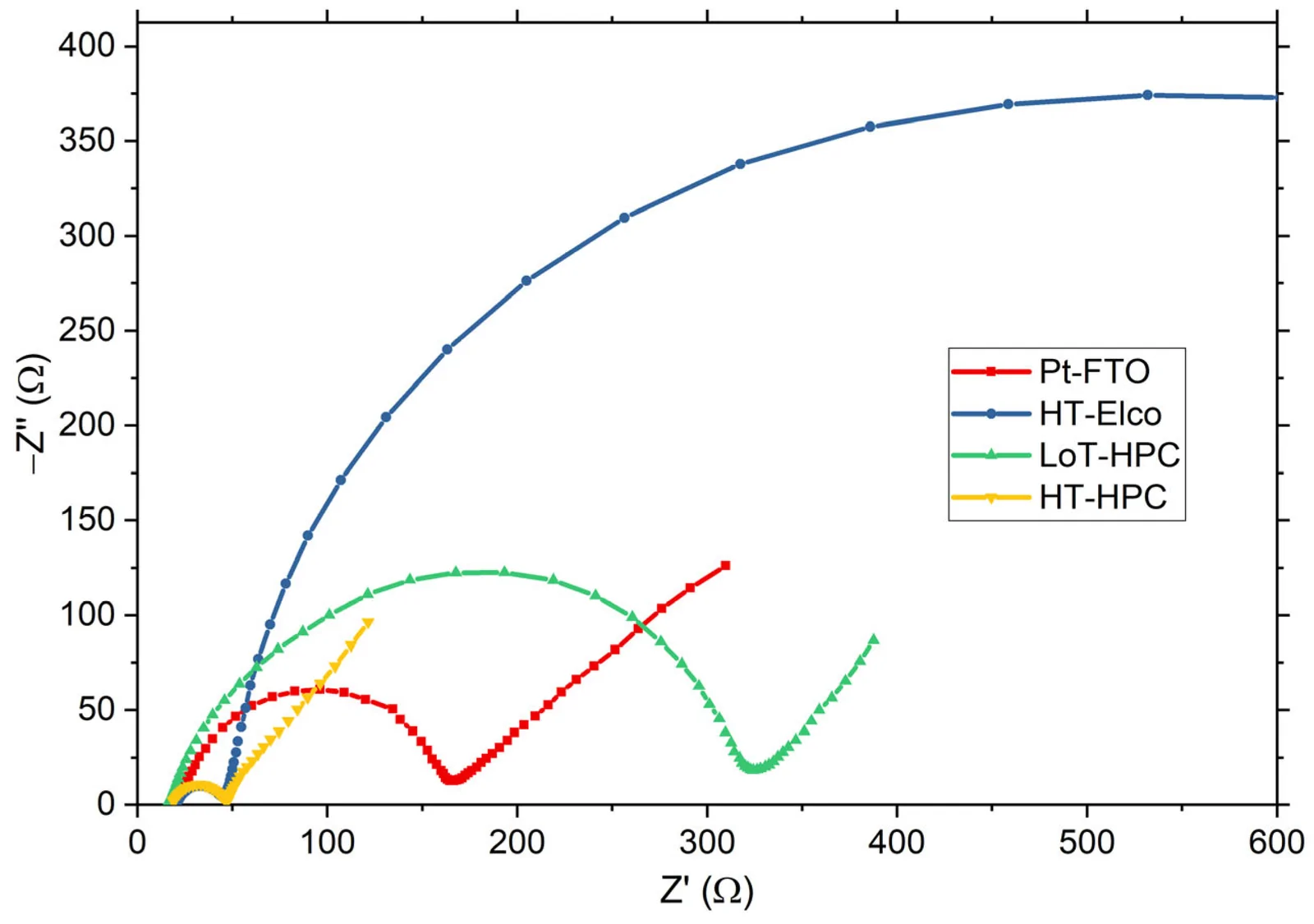
Electrochemical impedance spectroscopy in half-cell configuration of the reference Pt-FTO, the commercial HT-Elco, the custom LoT-HPC electrode and the custom electrode treated for 2 h at 250 °C in vacuum (HT-HPC), evaluated in a three-electrode configuration. All measurements were conducted in a dilute iodide/triiodide electrolyte (10 mM NaI, 1 mM I_2_, 0.1 M NaClO_4_ in acetonitrile) employing a Ag/Ag+ reference electrode and a Pt spring counter electrode.

To confirm these observations without the interference of potential sweeping, steady-state EIS was performed. The Nyquist Plot is reported in Figure 4, while the Bode (modulus and phase) plots are reported in Figure S2a,b. In Figure S2, the equivalent circuits used to fit the single-electrode EIS spectra are also reported. The series resistance (R_s_) values extracted from the high-frequency real-axis intercept are consistent across all electrodes, ranging from 16.4 to 21.7 Ω, which confirms reproducible cell assembly. The Nyquist plot for the Pt-FTO electrode was modelled using a standard Randles equivalent circuit, comprising R_s_, the charge-transfer resistance (R_ct_), the interfacial capacitance (CPE_ct_), and a standard Warburg element representing semi-infinite linear diffusion (W_d_). Under the dilute electrolyte conditions of the cell, the planar Pt-FTO electrode exhibits a relatively high R_ct_ of 33.6 Ω. As recently elucidated in the literature regarding platinum-iodide systems, such elevated charge-transfer resistance values are physically expected when operating in the millimolar concentration regime, where the exchange current density is significantly reduced by the limited availability of the active redox species [33].

**Figure 5:**
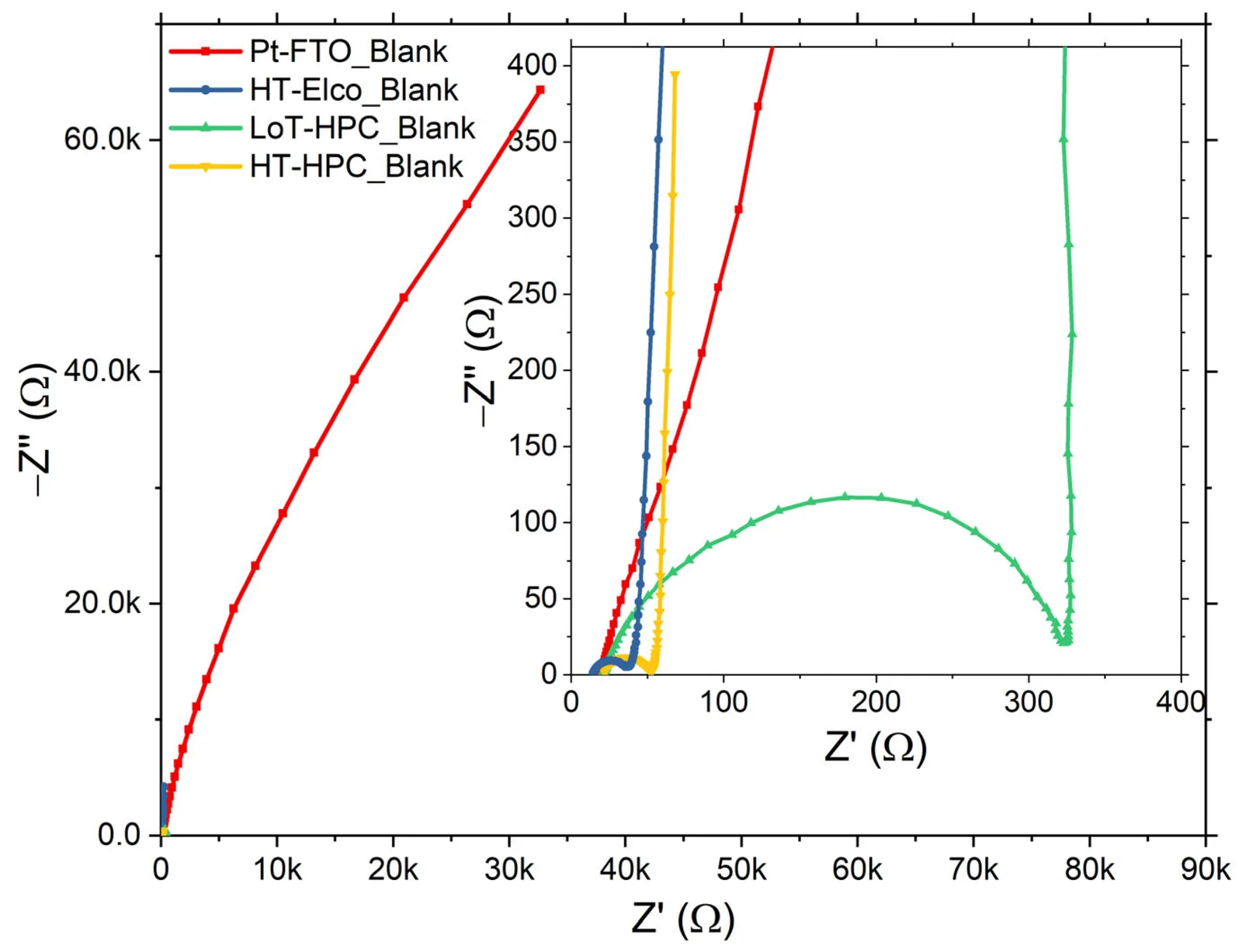
Electrochemical impedance spectroscopy in half-cell configuration of the reference Pt-FTO, the commercial HT-Elco, the custom LoT-HPC electrode and the custom electrode treated for 2 h at 250 °C in vacuum (HT-HPC), evaluated in a three-electrode configuration. All measurements were conducted in a blank electrolyte (0.1 M NaClO_4_ in acetonitrile) employing an Ag/Ag+ reference electrode and a Pt spring counter electrode.

In contrast, a different equivalent circuit configuration was required to model the carbon-based electrodes. For the commercial HT-Elco electrode, the Nyquist plot in the presence of the redox mediator exhibits two distinct semicircles, located in the high-frequency and medium-to-low-frequency regions, respectively. The impedance spectrum of the LT-HPC electrode, instead, qualitatively resembles the single-semicircle profile observed for the Pt-FTO electrode. To gain a deeper understanding of the physical processes underlying these features in the presence of the redox mediator, EIS measurements were carried out in a blank, redox-free supporting electrolyte (0.1 M NaClO_4_ in acetonitrile). The resulting non-faradaic Nyquist plots for the four electrodes are illustrated in Figure 5.

In the absence of redox-active species, both carbonaceous electrodes exhibit a well-defined high-frequency semicircle, shifted from the origin by the series resistance R_s_, followed by a nearly vertical capacitive line at low frequencies. Under these non-faradaic conditions, this high-frequency feature is widely attributed to the electronic charge percolation pathways, inter-particle contact resistance within the porous carbonaceous network, and the contact impedance between the active material film and the FTO current collector [13,34]. The percolation resistance (R_p_) associated with this high-frequency loop is remarkably low for the sintered HT-Elco electrode (24.9 Ω), while it is significantly larger for the LT-HPC electrode (312.6 Ω). This difference is highly consistent with the structural and composition profiles of the two films. Sintered at 400 °C, the commercial HT-Elco film is completely free of both the binder and the terpineol solvent. This yields a highly compact and electrically conductive carbon matrix, which greatly facilitates charge percolation.

In contrast, the non-fired LT-HPC electrode exhibits a much more difficult charge percolation pathway, which can be attributed to the insulating nature of the trapped terpineol solvent molecules. This resistive limitation is in perfect agreement with the three-electrode cyclic voltammetry results, which showed a highly resistive behaviour and poor electrochemical reversibility. To validate this hypothesis, the impedance spectrum of the vacuum-treated HT-HPC electrode (fired at 250 °C) was also recorded (Figure 5). The complete thermal evacuation of the residual terpineol yields a dramatic improvement in charge transport, with the percolation resistance dropping to just 27.8 Ω, a value highly comparable to that of the commercial HT-Elco benchmark. This dramatic reduction in internal resistance is also in excellent agreement with the improved reversibility and significantly narrower ΔE_p_ observed in the CV measurements for the HT-HPC sample. Finally, it is worth noting that this high-frequency semicircle is completely absent in the impedance spectrum of the Pt-FTO electrode under the same non-redox conditions, which displays only a standard series resistance coupled with a purely capacitive vertical line. This further confirms that the high-frequency loop is a unique structural fingerprint of the three-dimensional porous carbon network and is not related to any interfacial faradaic process, in this case.

After introducing the active I^−^/I_3_^−^ redox couple into the electrolyte, the high-frequency semicircle remains almost unaltered, confirming its attribution to the solid-state charge percolation and contact resistances of the carbon network (Figure 4). The main modification is observed in the low-frequency region, where the vertical, blocking capacitive line characteristic of the non-faradaic background is depressed, giving rise to a second semicircle (or a distorted arc) due to the activation of the faradaic charge-transfer reaction at the pore walls, which makes the interface non-blocking. Driven by these observations, the equivalent circuit model for the porous carbon electrodes was finally structured as illustrated in Figure S2c. Apart from the series resistance R_s_, the high-frequency R_p_//CPE_p_ parallel loop accounts for the electronic percolation and contact resistance within the 3D carbon matrix, and the low-frequency parallel combination includes the double-layer constant phase element (CPE) in parallel with the faradaic branch consisting of the charge-transfer resistance (R_ct_) and the restricted linear Warburg diffusion impedance (W). This modelling approach is similar to several studies in the literature that employ dual-loop equivalent circuits to capture the distinct contributions of pore resistance, contact resistance, and interfacial charge transfer in carbon-based counter electrodes for DSSCs [21,35,36]. However, these works typically present the fit on the active redox system directly, without validating the high-frequency percolation loop through a corresponding blank, redox-free electrolyte characterization.

Based on this fitting methodology, the commercial HT-Elco electrode exhibits a very high R_ct_ of 840 Ω, which is significantly larger than that of the Pt-FTO baseline (33.6 Ω). This resistive behaviour is consistent with the three-electrode cyclic voltammetry results. Indeed, despite the mesoporous nature of the graphite/carbon black matrix, which should theoretically provide a much larger electroactive surface area compared to the non-porous Pt-FTO electrode, the peak faradaic current densities for HT-Elco do not scale proportionally and remain comparable to or lower than those of Pt, reflecting a lower intrinsic electrocatalytic activity of the bare carbon surfaces for the triiodide reduction reaction. Regarding the custom active carbon electrodes, the LoT-HPC and HT-HPC films display significantly lower charge-transfer resistances of 305.7 Ω and 294.1 Ω, respectively. Firing the custom carbon film at 250 °C for HT-HPC yields a slight decrease in R_ct_, indicating a minor enhancement in the interfacial electrocatalytic kinetics after complete terpineol removal. This moderate improvement is in excellent agreement with the cyclic voltammetry curves (Figure 3), where only a modest increase in the cathodic triiodide reduction peak current is observed after vacuum-thermal treatment.

Finally, an additional investigation was performed to further understand the electrochemical behaviour of the prepared active carbon counter electrode. Specifically, the response of the impedance spectrum was evaluated after varying the concentration of the redox mediator. Figure S3 compares the Nyquist plots of a custom HT-HPC electrode recorded under three distinct electrolyte concentrations (all formulated in acetonitrile to ensure consistency): the baseline dilute electrolyte (10 mM NaI, 1 mM I_2_, and 0.1 M NaClO_4_), an intermediate concentration electrolyte (0.1 M NaI, 1 mM I_2_, and 0.1 M NaClO_4_), and the high-concentration formulation optimized for DSSC integration (0.45 M NaI and 0.055 M I_2_). As shown by the EIS spectra, while the overall geometric shape of the impedance profile remains qualitatively identical, both the high-frequency percolation resistance and the charge-transfer resistance decrease progressively with increasing ionic concentration. This trend strongly validates the concentration dependence of both charge-transport and interfacial charge-transfer processes. On one hand, according to Butler–Volmer kinetics, the exchange current density J_0_ is directly proportional to the concentration of the electroactive species [20,37]. Since R_ct_ is inversely proportional to J_0_, the increase in iodide and triiodide concentrations directly drives up the interfacial reaction rate, leading to a drop in the charge-transfer resistance.

On the other hand, the decrease in percolation resistance after increasing the electrolyte concentration may be consistent with a field-assisted electronic transport mechanism within the porous carbonaceous network. According to double-layer theory, the increased ionic strength drives a compression of the Debye screening length at the sub-nanometric inter-particle junctions, narrowing and lowering the electrostatic potential barriers that electrons must overcome to hop from one carbon grain to the next [31,38]. Then, a higher concentration of highly polarizable iodide anions (I^−^) may promote strong specific adsorption on the carbon active sites [31,39]. This adsorption could act as a local electrostatic dopant, increasing the electronic charge carrier density at the carbon grain boundaries. Therefore, the electronic charge percolation pathways would be optimized, lowering physical and resistive bottlenecks imposed by the insulating terpineol residue. Nevertheless, a comprehensive investigation into the microscopic coupling of these interfacial dynamics remains beyond the scope of the present work and is left to future research, where advanced transmission-line modelling will be systematically addressed.

In conclusion, the half-cell electrochemical characterizations (CV and EIS) confirm that while the Pt-FTO electrode presents the highest intrinsic catalytic activity, the 3D porous carbonaceous networks successfully compensate for their slower charge-transfer kinetics by offering a much higher electroactive surface area. Sintering of the custom carbon films (HT-HPC) effectively eliminates solvent-trapping bottlenecks, drastically reducing electronic percolation resistance and optimizing pore accessibility. To evaluate how these factors interact under actual device conditions, the next section explores the photovoltaic performance of these counter electrodes integrated within fully operational dye-sensitized solar cells (DSSCs).

### 3.3. Dye-Sensitized Solar Cell Characterization

**Figure 6:**
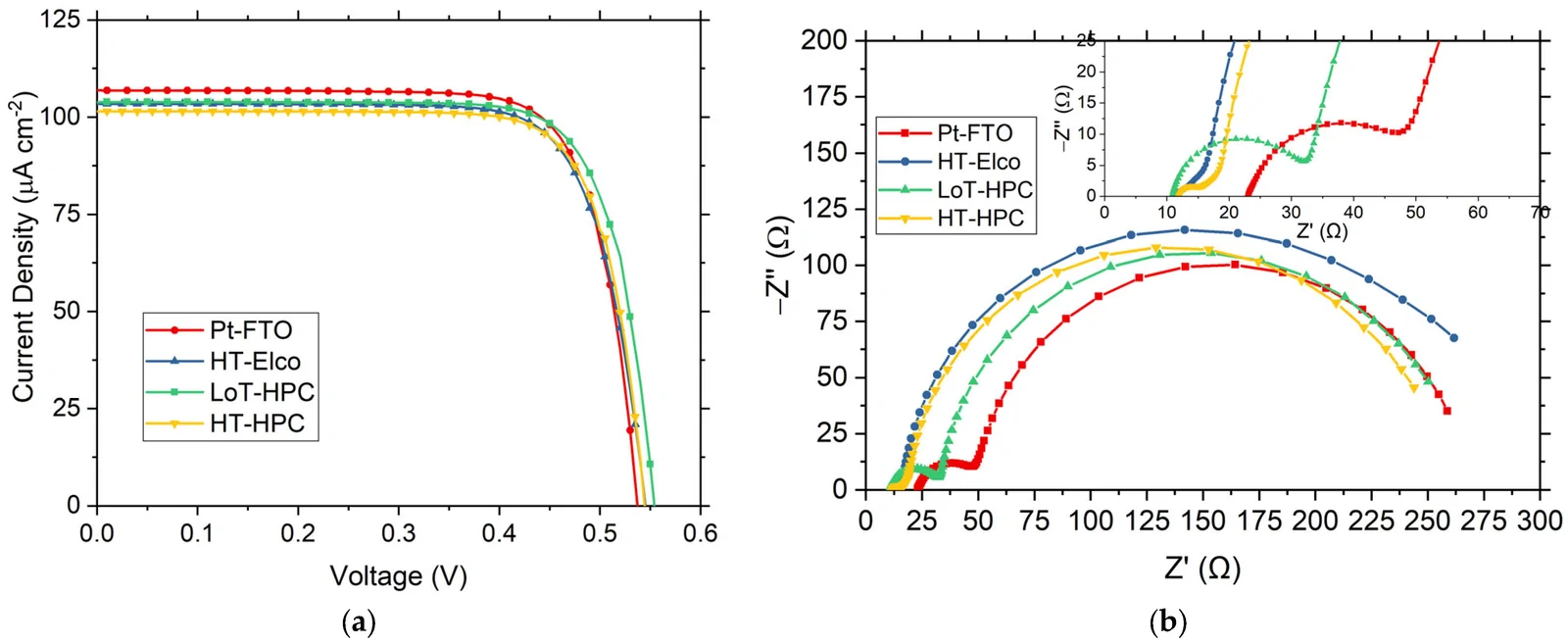
DSSC photovoltaic and electrochemical impedance characterization. (**a**) Current density–voltage (J-V) curves of the DSSCs employing the reference Pt-FTO, the commercial HT-Elco, and the custom LoT-HPC, HT-HPC counter electrodes, recorded under 1000 lux indoor illumination (0.3 mW cm^−2^). (**b**) Corresponding Nyquist plots obtained from full-cell EIS measurements conducted under 1000 lux at a constant bias equal to the device V_oc_.

To evaluate the practical applicability of the engineered counter electrodes under artificial light harvesting conditions, full DSSCs were assembled and characterized under varying indoor illumination intensities (100, 500, and 1000 lux). The photovoltaic parameters display excellent scaling with the illuminance level (Figure S4). The three devices exhibit highly comparable overall performances, with the minor variations falling well within standard experimental variability. Under 1000 lux (Figure 6a and Table 2), the DSSC including the custom LoT-HPC counter electrode achieves a PCE of 14.8% (J_sc_ = 103.9 µA cm^−2^, V_oc_ = 0.55 V, FF = 0.77). This performance is perfectly in line with, and even marginally superior to, the reference cells based on commercial HT-Elco paste (PCE = 14.2%, J_sc_ = 103.4 µA cm^−2^, V_oc_ = 0.55 V, FF = 0.76) and the standard Pt-FTO electrode (PCE = 14.7%, J_sc_ = 106.8 µA cm^−2^, V_oc_ = 0.54 V, FF = 0.77). Interestingly enough, there is virtually no difference from the DSSC prepared with the HT-HPC electrode, which showed a PCE of 14.3% (J_sc_ = 101.5, Voc = 0.54, FF = 0.77). The same high degree of similarity in device metrics is consistently observed at lower illuminance values (500 lux and 100 lux) (Figure S4). These results clearly demonstrate that under ultra-low indoor illuminance levels, any counter electrode possessing a minimum threshold of electrocatalytic activity is fully capable of sustaining the micro-ampere-scale photocurrents (ranging from 10 to 110 µA cm^−2^) generated at the photoanode of the DSSCs. Therefore, the severe kinetic and charge-transport limitations observed for the carbonaceous electrodes during half-cell three-electrode characterizations (such as high charge-transfer and percolation resistances) become negligible under indoor operating conditions. This behaviour successfully reproduces key findings from the literature, highlighting that indoor light environments are unique and fundamentally different from standard outdoor 1-sun conditions. Specifically, because the low photon flux reduces the photogenerated current density by approximately two to three orders of magnitude, the severe requirements on counter electrode catalytic kinetics and electrolyte ionic diffusion are drastically relaxed [20].

To further investigate the interfacial charge-transfer dynamics and correlate them with the observed photovoltaic parameters under working conditions, full-cell EIS was performed under 1000 lux constant LED illumination, applying a constant-voltage bias equal to the device V_oc_ (Figure 6b).

A preliminary qualitative analysis of the Nyquist plots reveals that the medium-to-low-frequency region is almost identical for all investigated devices. This behaviour is consistent since it is known that the medium-frequency arc in DSSC impedance spectra is governed by the electron recombination resistance (R_rec_) and the chemical capacitance (C_µ_) at the electrolyte-photoanode interface [40]. Since the photoanode architecture and the photogeneration rate under 1000 lux are kept identical across all tested cells, the back-electron transfer kinetics should remain unaltered and are not influenced by the nature of the counter electrode.

The high-frequency region, instead, displays clear differences among the devices, although its physical interpretation could be misleading. For the carbon-based counter electrodes, the individual contributions of the solid-state charge percolation (R_p_) and the interfacial faradaic charge-transfer resistance (R_ct_) cannot be clearly decoupled, representing a major challenge compared to the planar Pt-FTO reference. The commercial HT-Elco device exhibits a very tiny high-frequency feature followed by an inclined line. Based on the observation previously made during the three-electrode measurements, and because the exact nature of this combined response is not fully resolved under working device conditions, attempting to perform a numerical fit to extract precise resistance values would be physically misleading.

For the custom carbon electrodes, a significant reduction in the high-frequency semicircle is clearly visible when passing from the low-temperature (LoT-HPC) to the vacuum-temperature-treated (HT-HPC) counter electrode. This trend is highly consistent with our previous three-electrode half-cell and blank characterizations. On one hand, the removal of the insulating terpineol solvent improves electronic percolation. On the other hand, the removal of this solvent barrier slightly enhances the faradaic charge-transfer kinetics. Mathematically, when these two physical phenomena exhibit highly similar characteristic time constants (τ = R × C), their respective impedance contributions may overlap in the high-frequency domain, merging into a single convoluted semicircle that cannot be resolved into individual components [41,42].

It must be noted that the complete DSSC devices in this study were fabricated using γ-valerolactone (γ-VL) as a green, low-toxicity electrolyte solvent, rather than acetonitrile. The higher viscosity and different coordinating ability of γ-VL compared to volatile acetonitrile explain why the platinized reference electrode (Pt-FTO) exhibits a R_ct_ of approximately 24 Ω. This value is in excellent agreement with the findings reported in our previous work on γ-VL-based DSSCs, where the Pt/charge-transfer kinetics were shown to be limited by the viscous properties and different solvating shells of the green solvent compared to volatile acetonitrile [20].

Finally, these findings highlight the need for extreme caution when analyzing the impedance spectra of DSSCs employing porous carbonaceous counter electrodes. Neglecting the high-frequency charge percolation within the thick carbon matrix, or treating the entire high-frequency loop as a simple interfacial charge-transfer resistance, may represent a critical oversimplification which can lead to flawed interpretations and overestimated electrocatalytic performance in the literature.

A more comprehensive and quantitative investigation of these coupled electronic-ionic transport mechanisms within sub-nanometric pore networks is beyond the scope of this study and is left to future work. The primary objective of the present study is to demonstrate that the custom LT-HPC formulation, despite the bottlenecks coming from the residual terpineol solvent, can deliver photovoltaic performance under indoor light fully comparable to that of the reference systems (commercial HT-Elco and Pt-FTO). This result was achieved while introducing significant industrial and environmental advantages, such as the elimination of the energy-intensive, high-temperature firing step (400 °C) required for commercial carbon pastes like HT-Elco and the avoidance of the need for platinum, which remains a scarce, expensive, and critical raw material.

To validate the practical viability of this low-temperature approach, the final phase of this study investigated the long-term electrochemical and chemical stability of the custom LT-HPC counter electrode integrated within fully operational DSSCs. Specifically, it was essential to verify whether the trapped terpineol residues could trigger degradation pathways, lead to leakage, or passivate the active materials over prolonged operating times. To assess this, a continuous light-soaking and electrochemical stress test was carried out under a constant LED illumination at 1000 lux. The LT-HPC-based DSSC was subjected to continuous current-voltage (J-V) cycling, performing consecutive forward and reverse scans at a slow scan rate of 1 mV/s to monitor potential hysteresis and charge accumulation. This scanning protocol was systematically repeated 550 times, representing approximately 200 h of continuous, uninterrupted illumination and active device operation.

Remarkable stability was observed for the custom LT-HPC-based device, with the power conversion efficiency remaining virtually unchanged, transitioning from an initial 14.8% to a final value of 14.6% after the continuous 200 h light-soaking and cycling test (Figure S5). This exceptional performance retention is characterized by a minor decrease in the J_sc_, which slightly decreased from 103.9 to 97.8 µA cm^−2^, which was compensated by an increase in the V_oc_ from 0.55 to 0.57 V. Meanwhile, the FF remained practically constant, slightly rising from 0.77 to 0.78.

This outstanding stability provides a strong indication of robust electrochemical and mechanical integrity of the custom LT-HPC counter electrode under operational conditions. In DSSCs, the FF is heavily affected by the internal series resistance and the counter electrode’s charge-transfer resistance [35,43]. Therefore, any structural degradation, delamination of the carbon film, or progressive chemical passivation of the active catalytic sites would manifest as a severe, continuous drop in the FF [42]. The near-perfect preservation of the FF over 200 h of continuous active operation confirms that the electrical percolation networks within the custom 3D active carbon matrix remain exceptionally stable, and that the trapped terpineol residues do not induce any progressive deterioration or passivation of the catalytic active sites. Furthermore, the minor drop in J_sc_ can be ascribed to typical, slow degradation of the dye or slight electrolyte evaporation typical of long-term ageing, rather than counter electrode degradation, while the marginal increase in V_oc_ points toward a favourable, slow shift in the TiO_2_ conduction band or a reduction in back-electron recombination at the photoanode/electrolyte interface, possibly assisted by slow reorganizations within the γ-valerolactone coordinating shell [20,44]. Overall, these results demonstrate that the low-temperature-processed LT-HPC electrode represents a highly stable, cost-effective, and platinum-free alternative, capable of ensuring durable operation for indoor DSSC applications.

## 4. Conclusions

In this study, a highly sustainable, fluorine-free, and low-temperature screen-printable carbon composite (LoT-HPC) was successfully developed and validated as a highly efficient counter electrode for indoor dye-sensitized solar cells (DSSCs). By using only 5 wt% of the bio-derived hydroxypropyl cellulose (HPC) as a binder, the custom ink entirely bypasses the energy-intensive thermal treatments typically required by commercial carbon pastes (e.g., 400 °C for 40 min), requiring only a mild drying step at 100 °C for 15 min.

Electrochemical half-cell characterizations demonstrated that the highly active surface area of the custom carbonaceous network successfully compensates for the inherently lower intrinsic catalytic activity of bare carbon compared to platinum. The HPC-based formulation preserves the three-dimensional porous network of the activated carbon/carbon black composite, ensuring a high density of electrochemically accessible active sites. Half-cell cyclic voltammetry and electrochemical impedance spectroscopy (EIS) revealed that while the low-temperature-processed LoT-HPC electrode suffers from a solid-state charge percolation bottleneck and a widened peak-to-peak due to the trapped terpineol solvent, this transport limitation is dramatically resolved upon vacuum-thermal (250 °C) evacuation in the HT-HPC electrode, with the percolation resistance dropping to much lower values. Crucially, the operating device physics under dim indoor light reveals that the micro-ampere-scale current densities drastically relax the kinetic and charge-transport demands of the system, making the percolation and transfer bottlenecks completely harmless during actual indoor device operation.

Indeed, when integrated into full DSSC devices and tested under dim indoor illumination 1000 lux, the LoT-HPC custom device delivered a power conversion efficiency of 14.8% and a short-circuit current density of 103.9 µA cm^−2^, a photovoltaic performance highly comparable to that of standard sputtered Pt-FTO (14.7%) and commercial HT-Elco (14.2%) references. Furthermore, continuous light-soaking and J-V cycling under 1000 lux for 200 h demonstrated the exceptional operational stability of the custom DSSC, with the PCE experiencing a negligible decrease from 14.8% to 14.6%. This long-term robustness indicates that the trapped terpineol residues do not trigger degradation pathways or passivate the catalytic sites. Ultimately, these findings prove that the strategic integration of the HPC binder with a highly porous carbon matrix provides a scalable, energy-efficient, and sustainable alternative to conventional platinum and high-temperature carbon pastes, fulfilling the economic demands of indoor photovoltaics for Internet of Things (IoT) applications.

## Figures and Tables

**Table 1:** Relationship between the number of printed layers, dry film thickness, and sheet resistance for the custom low-temperature (LoT-HPC) and commercial high-temperature (HT-Elco) carbon counter electrodes.

	LoT-HPC		HT-Elco	
# of Printed Layers	Thickness (µm)	Sheet Resistance (Ω/sq)	Thickness (µm)	Sheet Resistance (Ω/sq)
1	29 ± 3	813 ± 5	16 ± 1	24 ± 3
2	55 ± 6	484 ± 8	30 ± 2	10 ± 2
3	70 ± 7	206 ± 7	43 ± 1	8 ± 1

**Table 2:** Photovoltaic parameters of the DSSCs fabricated with the different electrodes recorded at 1000 lux (0.3 mW cm^−2^).

	Jsc (µA cm^−2^)	Voc (V)	FF	η (%)
Pt-FTO	106.8	0.54	0.77	14.7
HT-Elco	103.4	0.55	0.76	14.2
LoT-HPC	103.9	0.55	0.77	14.8
HT-HPC	101.5	0.54	0.77	14.3

## Data Availability

Data are available from the authors upon request.

## Supplementary Materials

The following supporting information can be downloaded at: https://www.mdpi.com/article/10.3390/nano16161007/s1, Figure S1. (a) Amplitude sweep test of the LoT-HPC and HT-Elco inks. (b) Thermogravimetric curves of the inks among 25–800 °C. Figure S2. Bode plots obtained from electrochemical impedance spectroscopy (EIS) measurements of the standard Pt-FTO, the commercial HT-Elco, the custom LoT-HPC and the treated custom electrode HT-HPC, evaluated in a three-electrode half-cell configuration together with the equivalent circuit to fit the impedance spectra. Figure S3. Bode plots obtained from electrochemical impedance spectroscopy (EIS) measurements of the standard Pt-FTO, the commercial HT-Elco, and the custom LoT-HPC electrodes evaluated in a three-electrode half-cell configuration. Figure S4. Evolution of the photovoltaic parameters for the DSSCs fabricated with the reference Pt-FTO, the commercial HT-Elco, the custom LoT-HPC counter electrode and the same treated at high temperature HT-HPC, evaluated under varying indoor illumination intensities (100, 500, and 1000 lux). Table S1. Photovoltaic parameters for the DSSCs fabricated with the reference Pt-FTO, the commercial HT-Elco, the custom LoT-HPC counter electrode and the same treated at high temperature HT-HPC, evaluated under varying indoor illumination intensities (100, 500, and 1000 lux). Figure S5. Long term stability of the photovoltaic parameters for the DSSCs fabricated with the custom LoT-HPC counter electrode under continuous illumination of 1000 lux and cyclic forward and reverse polarization at 1 mV s^−1^. Figure S6. N_2_ adsorption-desorption isotherms of LoT-HPC ink before and after the thermal treatment. Inset: Pores distribution by using the NLDFT approach. Table S2. N_2_ adsorption-desorption data. Micropore area and micropore volume have been computed by using t-plot, in particular the Harkins and Jura thickness curve and BET surface area in the range 0.35–0.5 nm.
