# Supplementary material for: Hydroxypropyl Cellulose as an Effective Binder for Low-Temperature Screen-Printed Porous Carbon Counter Electrodes for Indoor Dye-Sensitized Solar Cells
Source: Nanomaterials (Basel). 2026 Aug 17;16(16):1007. doi: 10.3390/nano16161007 (PMC13516536; doi:10.3390/nano16161007)
Supplement: Supplementary file 1 [file nanomaterials-16-01007-s001.zip › nanomaterials-4454325-supplementary.pdf]

nanomaterials

Supplementary material

Hydroxypropyl Cellulose as an Effective Binder for Low-Temperature Screen-Printed Porous Carbon Counter Electrodes for Indoor Dye-Sensitized Solar Cells

Roberto Speranza[1,*], Elisa Morale[1], Filippo Sergiacomi[1], Angelica Bisceglie[1,2], Giorgio Mogli [1,2], Simone Martellone[1,2] and Andrea Lamberti [1,2]

[1] Department of Applied Science and Technologies, Politecnico di Torino , Corso Duca Degli Abruzzi 24, 10129 Torino, Italy; elisa.morale@studenti.polito.it (E.M.); filippo.sergiacomi@studenti.polito.it (F.S.); angelica.bisceglie@polito.it (A.B.); giorgio.mogli@polito.it (G.M.); simone.martellone@polito.it (S.M.); andrea.lamberti@polito.it (A.L.)
[2] Center for Sustainable Future Technologies, Istituto Italiano di Tecnologia, 10129 Torino, Italy
* Correspondence: roberto.speranza@polito.it

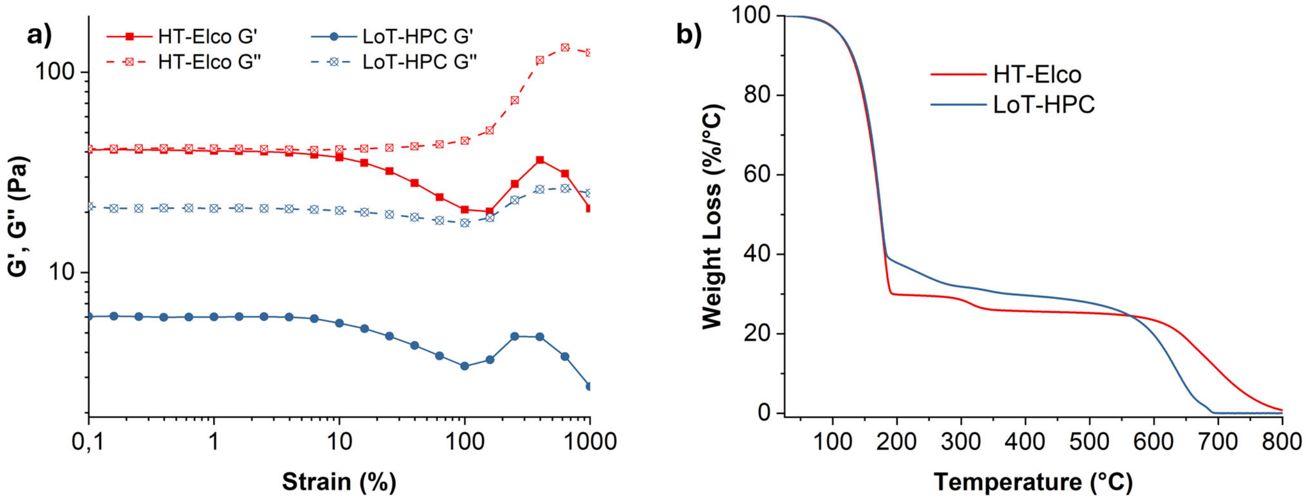

Figure S1. (**a**) Amplitude sweep test of the LoT-HPC and HT-Elco inks. (**b**) Thermogravimetric curves of the inks among 25–800°C.

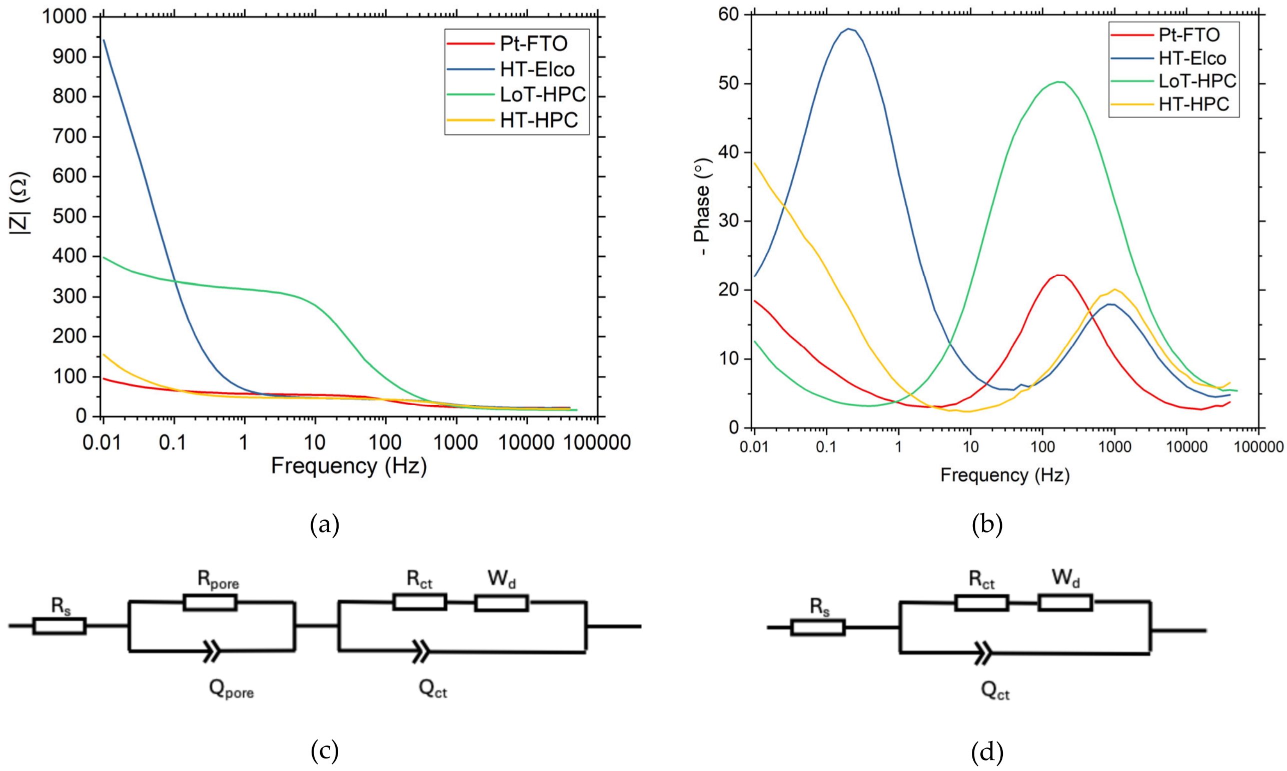

Figure S2. Bode plots obtained from electrochemical impedance spectroscopy (EIS) measurements of the standard Pt-FTO, the commercial HT-Elco, the custom LoT-HPC and the treated custom electrode HT-HPC, evaluated in a three-electrode half-cell configuration. (a) Impedance magnitude and (b) phase angle as a function of frequency. In c) is reported the equivalent circuit used to fit the impedance spectra of the carbon based electrodes while in (d) the one used to fit the spectra of the Pt-FTO electrode.

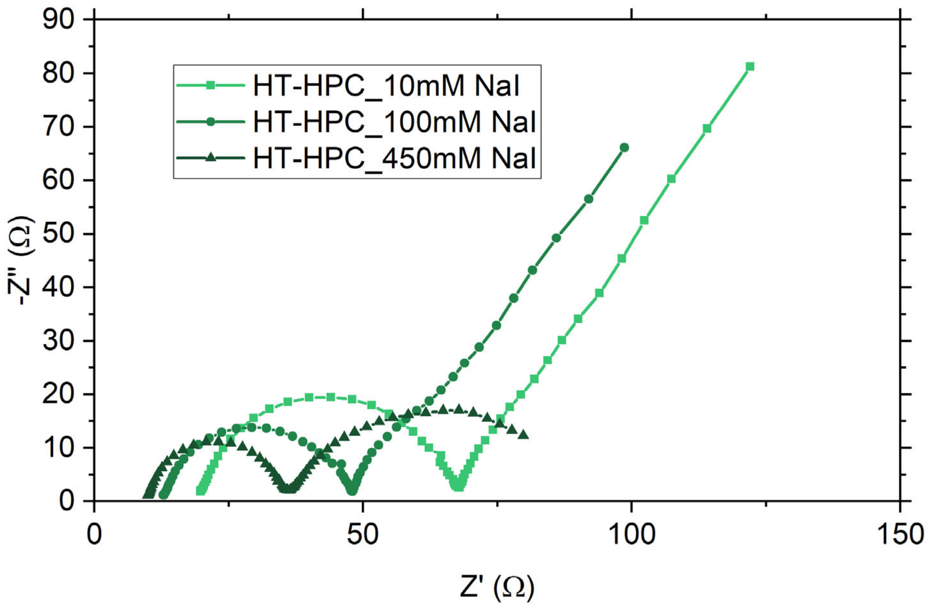

Figure S3. Electrochemical impedance spectroscopy in half-cell configuration of the the custom HT-HPC electrode evaluated in a three-electrode half-cell configuration under three distinct electrolyte concentrations (all formulated in acetonitrile to ensure consistency): the baseline dilute electrolyte (10 mM NaI, 1 mM I_2, and 0.1 M $NaClO_4$), an intermediate concentration electrolyte (0.1 M NaI, 1 mM I_2, and 0.1 M $NaClO_4$), and the high-concentration formulation optimized for DSSC integration (0.45 M NaI and 0.055 M I_2)

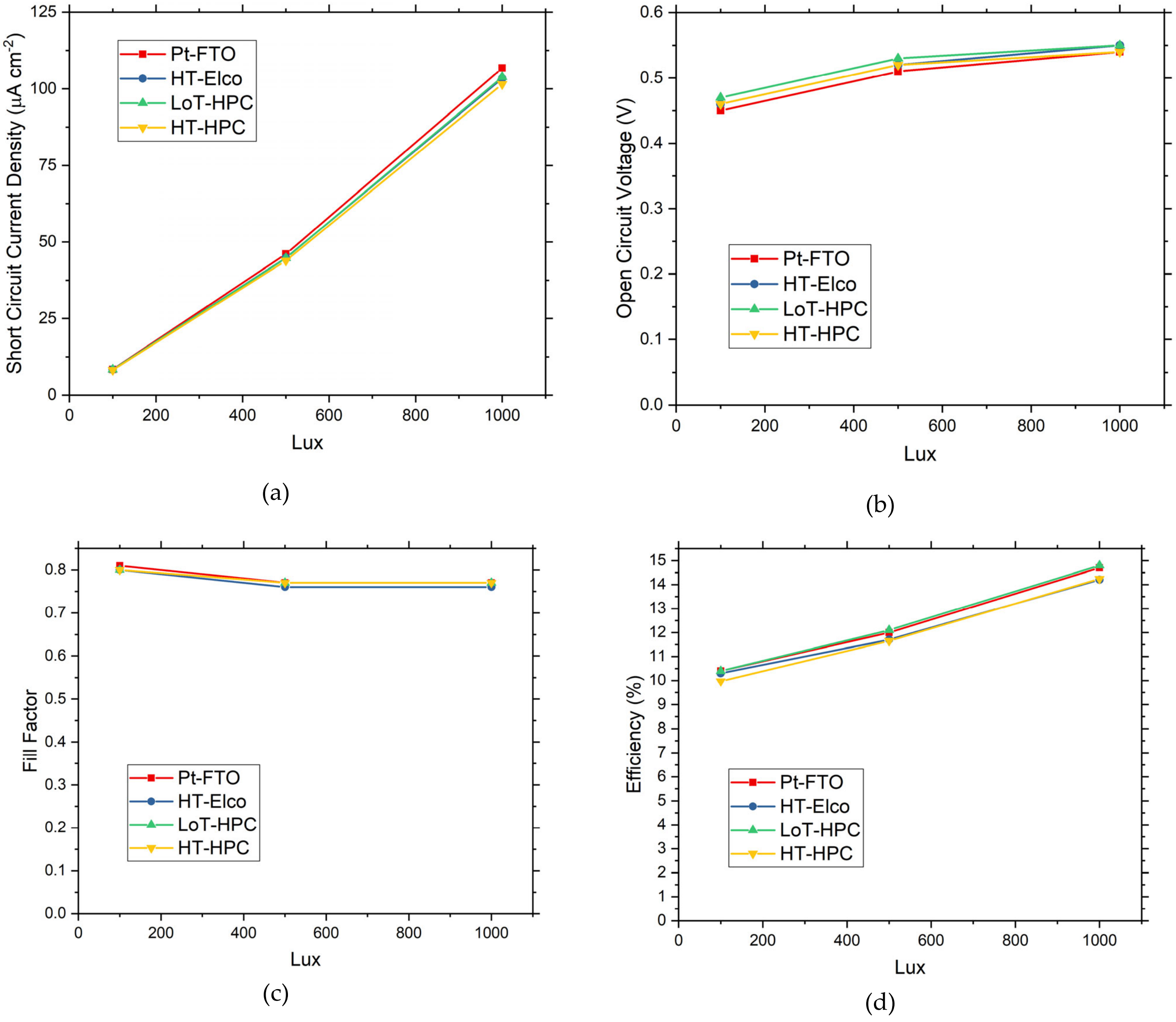

Figure S4. Evolution of the photovoltaic parameters for the DSSCs fabricated with the reference Pt-FTO, the commercial HT-Elco, the custom LoT-HPC counter electrode and the same treated at high temperature HT-HPC, evaluated under varying indoor illumination intensities (100, 500, and 1000 lux). (a) Short-circuit current density, (b) open-circuit voltage, (c) fill factor, and (d) power conversion efficiency.

https://doi.org/10.3390/nano16161007

Table S1. Photovoltaic parameters for the DSSCs fabricated with the reference Pt-FTO, the commercial HT-Elco, the custom LoT-HPC counter electrode and the same treated at high temperature HT-HPC, evaluated under varying indoor illumination intensities (100, 500, and 1000 lux).

	Lux	Jsc (μA cm^{-2})	Voc (V)	FF	η (%)
	100	8.4	0.45	0.81	10.4
**Pt-FTO**	500	46.1	0.51	0.77	12.0
	1000	106.8	0.54	0.77	14.7
	100	8.4	0.46	0.80	10.3
**HT-Elco**	500	44.8	0.52	0.76	11.7
	1000	103.4	0.55	0.76	14.2
	100	8.4	0.47	0.80	10.4
**LoT-HPC**	500	44.7	0.53	0.77	12.1
	1000	103.9	0.55	0.77	14.8
**HT-HPC**	100	8.2	0.46	0.80	10.0
	500	43.9	0.52	0.77	11.7
	1000	101.5	0.54	0.77	14.3

https://doi.org/10.3390/nano16161007

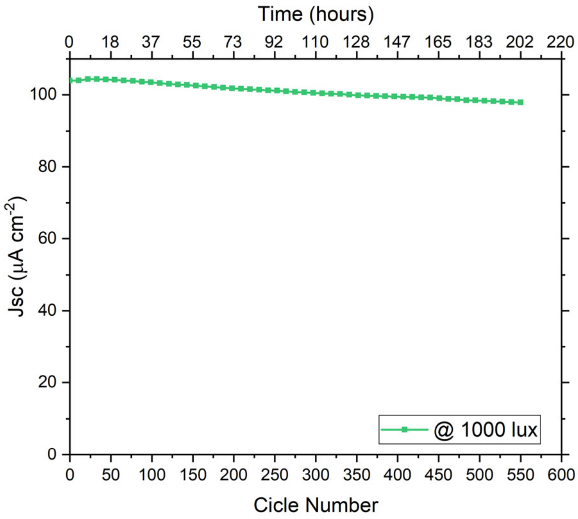

(a)

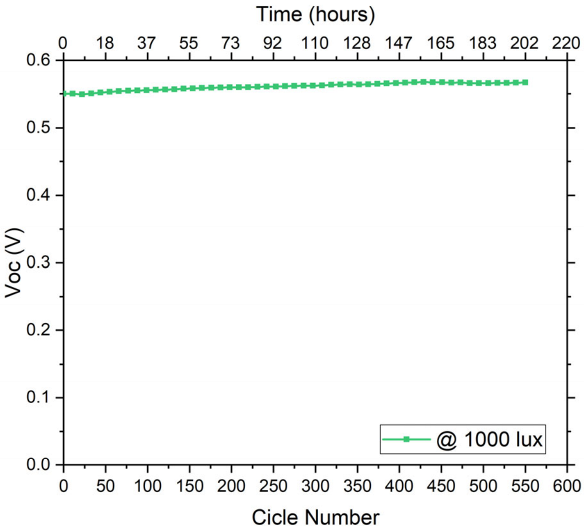

(b)

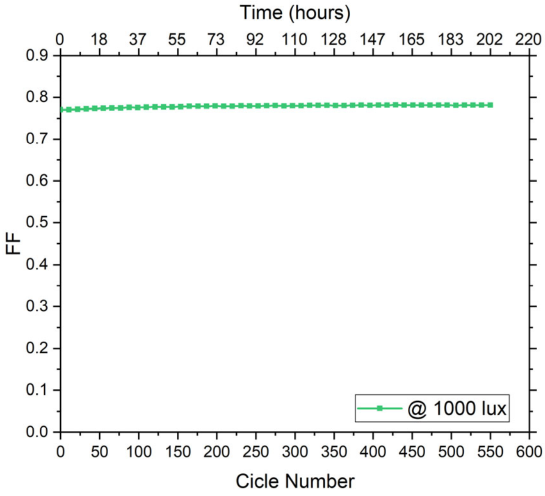

(c)

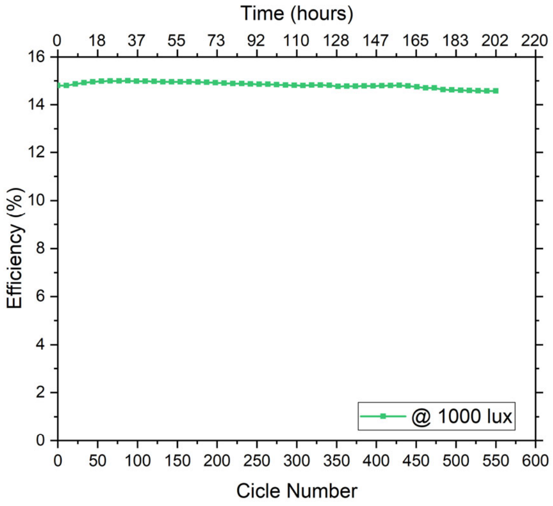

(d)

Figure S5. Long term stability of the photovoltaic parameters for the DSSCs fabricated with the custom LoT-HPC counter electrode under continuous illumination of 1000 lux and cyclic forward and reverse polarization at 1 mV s^{-1}. (a) Short-circuit current density, (b) open-circuit voltage, (c) fill factor, and (d) power conversion efficiency.

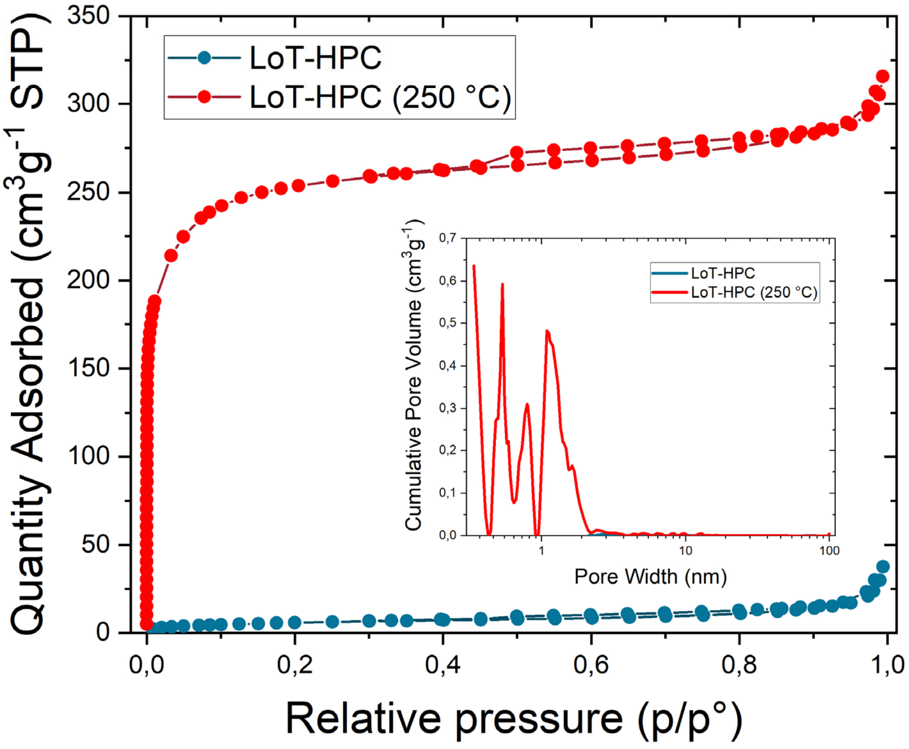

Figure S6. N_2 adsorption-desorption isotherms of LoT-HPC ink before and after the thermal treatment. Inset: Pores distribution by using the NLDFT approach.

Table S2. N_2 adsorption-desorption data. Micropore area and micropore volume have been computed by using t-plot, in particular the Harkins and Jura thickness curve and BET surface area in the range 0.35-0.5 nm.

Sample	BET m^2 g^{-1}	Micropore Area (m^2 g$^-$)	Micropore volume (cm^3 g^{-1})
**LoT-HPC**	21.9 ± 0.1		
**HT-HPC**	112.8 ± 4.9	844.9	0.3

https://doi.org/10.3390/nano16161007
